# Video game characteristics, happiness and flow as predictors of addiction among video game players: A pilot study

**DOI:** 10.1556/JBA.2.2013.005

**Published:** 2013-04-12

**Authors:** Damien C. Hull, Glenn A. Williams, Mark D. Griffiths

**Affiliations:** International Gaming Research Unit, Psychology Division, School of Social Sciences, Nottingham Trent University, Nottingham, United Kingdom

**Keywords:** video game addiction, structural characteristics of video games, happiness, flow

## Abstract

*Aims:* Video games provide opportunities for positive psychological experiences such as flow-like phenomena during play and general happiness that could be associated with gaming achievements. However, research has shown that specific features of game play may be associated with problematic behaviour associated with addiction-like experiences. The study was aimed at analysing whether certain structural characteristics of video games, flow, and global happiness could be predictive of video game addiction. *Method:* A total of 110 video game players were surveyed about a game they had recently played by using a 24-item checklist of structural characteristics, an adapted Flow State Scale, the Oxford Happiness Questionnaire, and the Game Addiction Scale. *Results:* The study revealed decreases in general happiness had the strongest role in predicting increases in gaming addiction. One of the nine factors of the flow experience was a significant predictor of gaming addiction – perceptions of time being altered during play. The structural characteristic that significantly predicted addiction was its social element with increased sociability being associated with higher levels of addictive-like experiences. Overall, the structural characteristics of video games, elements of the flow experience, and general happiness accounted for 49.2% of the total variance in Game Addiction Scale levels. *Conclusions:* Implications for interventions are discussed, particularly with regard to making players more aware of time passing and in capitalising on benefits of social features of video game play to guard against addictive-like tendencies among video game players.

## Introduction

Video game playing is prevalent across many cultures with a wide range of different types, genres, and interfaces from which to choose. These media have been subjected to an increasing number of studies concerning the adverse effects of video game play that may entail cases of video game addiction, for instance (Griffiths, Kuss & King, 2012; Griffiths & Meredith, 2009; Kuss & Griffiths, 2012a; 2012b). It has been argued that to be addicted to video games, six core components need to be experienced by the player (Griffiths, 2008), namely salience, mood modification, tolerance, withdrawal, conflict and relapse. *Salience* is evident when the playing of the video game becomes the single most important thing in a person's life, often resulting in cravings and total preoccupation with the activity. *Mood modification* involves the creation of an arousing (or, in some cases, a satiating) feeling from playing the game that is often used as a way of coping with other areas of the person's life. The mood-modifying effect from the game often requires increasing amounts of game time, leading to *tolerance*. When playing the game is not possible, players may experience *withdrawal* symptoms, including irritability, sweating, headaches, the shakes, etc. *Conflict* refers to the ways in which playing the game interferes with normal day-to-day life, compromising personal relationships, job and/or educational activities, and hobbies/social life. Players may also experience intra-psychic conflict (i.e. personal conflict, resulting in feelings of guilt and/or a loss of control). *Relapse* refers to the tendency for those attempting to change their behaviour to return to similar patterns of video game playing prior to stopping the last time around.

Addictive behaviours brought on by video game play could be elicited by positive psychological phenomena, such as the state of flow (Ting-Jui & Chih-Chen, 2003). With the flow experience, a game player derives intense enjoyment by being immersed in the gaming experience, the challenges of the game are matched by the player's skills, and the player's sense of time is distorted so that time passes without it being noticed (Csíkszentmihályi, 1992). For some video game players, this may then mean repeatedly seeking out similar experiences on a regular basis to the extent that they can escape from their concerns in the 'real world’ by being continually engrossed in a flow-inducing world (Sweetser & Wyeth, 2005). As can be seen, something like flow – viewed largely as a positive psychological phenomenon (Nakamura & Csíkszentmihályi, 2005) – may be less positive in the long-term for some video game players if they are craving the same kind of emotional ‘high’ that they obtained the last time that they experienced flow when playing a video game.

Flow has been proposed by Jackson and Eklund (2006) as comprising nine elements that include: (i) striking a balance between the challenges of an activity and one's abilities; (ii) a merging of performance of actions with one's self-awareness; (iii) possessing clear goals; (iv) gaining unambiguous feedback on performance; (v) having full concentration on the task in hand; (vi) experiencing a sense of being in control; (vii) losing any form of self-consciousness;(viii) having a sense of time distorted so that time seems to speed up or slow down; and (ix) the undergoing of an auto-telic experience (e.g., the goals are generated by the person and not for some anticipated future benefit).

The application of flow to video games is intuitive and some of the core concepts in gaming design indirectly incorporate facets of flow theory. For example, Dynamic Difficulty Adjustment (DDA) is a design implementation within a game that replaces the traditional, optional difficulty system (Hunicke & Chapman, 2004). Instead of deciding the difficulty level of the game from the offset, the difficulty setting is player-centred, offering modification based on the performance of the player. In this way, the game adjusts itself to keep players at the level of difficulty that is challenging them, while not distressing them. Chen (2007) created a game exclusively to demonstrate this feature, which incorporated a DDA system while discreetly informing players of their performance. The game had clearly set goals, and players reported that time seemed to ‘fly by’ when playing. The experience of time distortions is a common feature of gaming. Some studies (e.g. Wood & Griffiths, 2007; Wood, Griffiths and Parke, 2007) have obtained qualitative and quantitative data from players of video games to explore this issue. In one of their online surveys of 280 gamers (Wood et al., 2007), results showed that 99% of the gaming sample reported experiencing time loss at some point while playing video games. Further analysis showed that 17% experienced this occasionally, 49% frequently, and 33% all of the time. When performing an activity in a state of flow, if one suddenly becomes aware of one's self, this may result in ending the optimal experience (Csíkszentmihályi, 1992). For this reason, reports of losing track of time are often one of the best indications of flow-like experiences. However, in the case of Wood and Griffiths' (2007) study, time loss was not always reported as positive and such phenomena were often reported more negatively in terms of potential video game addiction.

**Figure 1.**
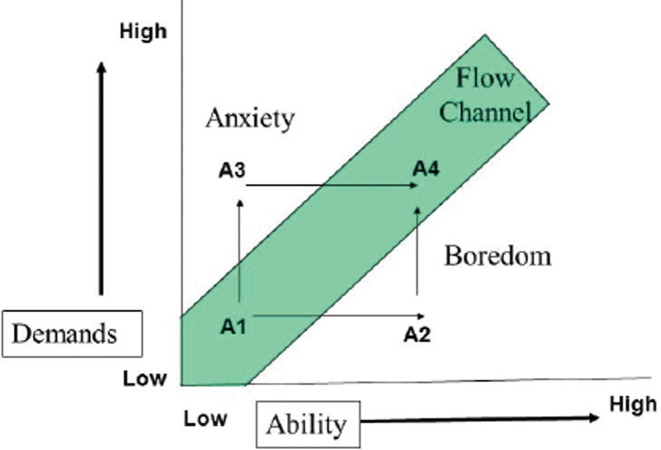
Flow in relation to video game play. Adapted from Csíkszentmihályi (1992)

Given that several studies have concentrated on psychological flow and addiction in relation to video games, it is not surprising that these two factors may at times inter-relate. Ting-Jui and Chih-Chen (2003) examined the relationship between flow and addiction and found that flow resulted from repetitive behaviour through a desire to repeat the positive experience. This repetitive behaviour subsequently resulted in addictive tendencies when wishing to repeat the activity concerned. It should be noted that not all players who experience flow may get addicted to playing a video game and not all persons who are addicted to video gaming will necessarily have a flow state when playing. Flow could be an antecedent to an increased likelihood of becoming addicted to a game as gamers begin to increase their expertise in playing the game and this may then lead to a seeking out of greater challenges within the game space to receive the same ‘hit’. For instance, as Figure 1 shows (adapted for video gaming), if the level of challenge is low and a player's abilities are low through just learning the game, there could be a flow-like experience as the player begins to revel in their new-found talents at a game (i.e. A1 in Figure 1). However, if the challenges of the game remain at a similar level throughout, then it is likely that the player may begin to get bored of the challenges of that game (i.e. A2 in Figure 1); by contrast, if the gamer were to be dropped into a level of a video game that was over-challenging for one's abilities (i.e. A3 in Figure 1), then anxiety may result and perhaps an inclination to no longer want to play the game. Where flow and addiction may begin to be intertwined is when the challenges of the game begin to increase in line with the player's abilities and new challenges need to be met. In this state (i.e. A4 in Figure 1), it has been argued (Csíkszentmihályi, 1992;p. 75) that this will be a more intense and complex flow experience markedly different from when the activity was first being learned. Due to the ‘highs' experienced at that point, it can be hypothesised that, as flow experiences increase in frequency through incremental steps of abilities matched with challenges when playing, it is likely that addiction-inducing situations may then begin to arise.

In addition to positive psychological phenomena (e.g. flow), being correlated with gaming addiction, there is evidence to suggest that low levels of well-being and happiness can be predictive of increased tendencies to engage in problematic video gaming behaviour. For instance, a two-wave longitudinal study by Lemmens, Valkenburg and Peter (2011) of 851 adolescents in The Netherlands found that poor states of psychological well-being acted as antecedents to pathological video gaming. Well-being, in that study, was operationalized in several forms that included self-esteem, social competence and loneliness. As a result of Lemmens et al.'s (2011) work, we predicted that low levels of happiness would be predictive of higher levels of gaming addiction scores.

Characteristic features that a video game may possess could impact on a player's experiences and the potential for a game to elicit addictive-type behaviours. To this end, King, Delfabbro and Griffiths (2010) developed a taxonomy of features and sub-features that are common to most video games (see Table 1). This taxonomy was based on the seminal work by Wood, Griffiths, Chappell and Davies (2004) who identified inherent characteristics of a video game that were structural and likely to induce initial gaming activities or maintenance of gaming, irrespective of any other differentiating factor such as the socioeconomic status, age, sex, and so forth. There has also been preliminary recent evidence (King, Delfabbro & Griffiths, 2011) to demonstrate that some structural features inherent in certain games are particularly associated with problematic behaviour that could pose a risk to players of having increased video game addiction tendencies. For example, King, Delfabbro and Griffiths (2011) found that video game players who were exhibiting problematic tendencies were more likely than so-called ‘normal’ players to look to games that provided increasing rewards in the game (e.g. earning experience points or finding rare items) and were also more likely to be engrossed in games with a high social component to them (e.g., sharing tips and strategies, cooperating with other players, etc.).

**Table 1.** Taxonomy of video game structural characteristics

Feature type	Sub-features	Example
Social features	Social utility features	In-game voice and text chat
	Social information/utility features	Guilds/clans in MMORPGs
	Leader board features	“Hall of fame” - high score list
	Support network features	Internet forums, strategy guides
Manipulation and control features	User input features	“Combos”, “hot keys“
	Save features	Checkpoints, “quicksave“
	Player management features	Managing multiple resources
	Non-controllable features	Scripted events, loading screens
Narrative and identity features	Avatar creation features	Choice of sex, race, attributes
	Story telling device features	Cut scenes, Mission briefing
	Theme and genre features	“Roleplaying”, “Shooting“
Reward features	General reward type features	Experience points, bonuses
	Meta-game reward features	Xbox 360 achievement points
	Intermittent reward features	Increasing difficulty or levels
	Payout interval features	Rewarded instantly for playing
Punishment features	Punishment features	Losing a life, restarting a level
	Negative reward features	Gaining health, repairing items
	Near miss features	Difficult “boss” at end of level
	Event frequency features	Unlimited replayability of game
	Event duration features	MMORPGs have no endpoint
Presentation features	Graphics and sound features	Realistic sounds, fast music
	Franchise features	Trade-marked names, e.g. Mario
	Explicit content features	Violence, drug use, nudity
	In-game advertising features	Real-life brands, sponsors, logos

*Note:* Adapted from King, Delfabbro and Griffiths (2010).

Overall, this pilot study was designed to test a predictive model of video game addiction that incorporated the structural characteristics of a game that participants had recently played, along with the nine different elements of flow that may have been experienced in relation to playing that game, along with the influence of respondents' general levels of happiness, or lack thereof.

It was hypothesised, based on prior research into flow and addiction, that flow would be positively associated with gaming addiction, in particular the elements of flow that would be symptomatic of being immersed in a state of being that would involve shutting out the player from the outside world (e.g. actions and self-awareness being blended into one; concentrating on the task; not being self-conscious; and having a sense of time being distorted). It was also anticipated that, as unhappiness has been correlated with tendencies to withdraw socially and be engrossed in activities such as excessive video gaming, we predicted that low levels of happiness would predict an increase in gaming addiction. Moreover, we expected to see positive associations between the main structural features identified by King, Delfabbro and Griffiths (2011) and gaming addiction, as per prior research. It was anticipated that the social features would affect a similar dynamic with addiction that has been seen previously in a range of virtual environments such as what may occur in social networking websites. Other features were also seen as synonymous in being able to elicit pleasurable engaged feelings of being in flow while at the same time leaving oneself open to such activities becoming addictive – these would entail the seeking out of rewards and the striving to be in control, to name a few of the structural characteristics that may be naturally reinforcing.

## Method

### Participants

A total of 190 gamers completed an online questionnaire. The sample was obtained via opportunity sampling by advertising through online gaming forums and via other online psychology research web sites. After data cleaning for incomplete or problematic responding (e.g., identical responses made for all items or illogical response patterns), a final sample comprising 110 responses was used. This included 78 males and 32 females, with a mean age of 24.7 years (*SD* = 9.04 years). The mean number of years of playing video games was 13.4 years (*SD* = 5.6 years) and participants played for a mean of 9.2 hours per week (*SD* = 8.8 hours). Respondents mainly came from the United States of America (*n* = 65) or the United Kingdom (*n* = 34). Overall, 79 different video games were played by the participants with the most common game being played by respondents being *Call of Duty: Modern Warfare 2* (*n* = 20). Of the 110 participants, 66 played the video game alone, while 42 played in a multiplayer mode. Two players did not specify whether they played alone or with others.

### Measures

*Video Game Features* (King et al., 2010). Respondents were provided with a selection of gameplay features, based on the video game feature taxonomy developed by King et al. (2010). Each item also included an example of what each kind of feature entailed (see sample items in Table 1). Gamers were asked to indicate the extent to which each of the characteristics was integral to the gaming enjoyment of the video game they had most recently played. Items were coded according to an ordinal scale of *2* if the feature was rated as *present and important*, *1* if the feature was *present but not important for gaming enjoyment* and *0* if it was *not present*. It should be noted that one of the five main features from the King, Delfabbro and Griffiths (2011) taxonomy – the reward/punishment one – was split into two and the focus of the analysis was primarily on the reward feature rather than on punishments as it was hypothesised that the seeking of rewards would be most strongly linked with gaming addiction and the avoidance of punishments would not be that crucial for gaming addiction levels.

*The Flow State Scale* (FSS-2; Jackson & Eklund, 2006). This was used to measure the degree of flow experienced while playing their indicated games. The FSS-2 is a 39-item scale assessing nine factors relating to flow, which were computed as subscales. Responses were scored on a 5-point Likert scale ranging from *strongly disagree* (1) to *strongly agree* (5). Higher scores for each of the nine factors indicated a strong indication of flow-like experiences having taken place. The scale has good psychometric properties, with confirmatory factor analysis supporting its factorial validity and it has satisfactory internal consistency, with Cronbach's alphas ranging from .72 to .91 (Jackson & Eklund, 2006).

*The Game Addiction Scale* (GAS; *Lemmens,* Valkenburg & Peter, 2009). The GAS was used to measure addiction relating to the game they had most recently played. The GAS is a 21-item scale, comprising seven subscales measuring factors of gaming addiction, and based on problematic behaviours and cognitions taken from the *Diagnostic and Statistical Manual of Mental Disorders* (American Psychiatric Association, 2000). The scale included questions such as: *Did you think about playing video games all day long?* that indicated the issue of *salience* in relation to addictive tendencies with video gaming. Responses were scored on a 5-point Likert scale, ranging from *never* (coded as *1*)to *very often* (coded as *5*). A score of *5* indicated a strong indication of addiction-proneness with regard to a specific factor. The Gaming Addiction Scale total was calculated by summing all items. The GAS has been found to have good levels of concurrent validity and very high internal consistency (Lemmens et al., 2009) and has been used in a range of studies into video game addiction (e.g., Arnesen, 2010; Hussain, Griffiths & Baguley, 2012; van Rooij, 2011).

*The Oxford Happiness Questionnaire* (OHQ; Hills & Argyle, 2002). The 29-item OHQ was used, which was a measure of general happiness. Items were scored on a 6-point Likert scale ranging from 1 = *strongly disagree* to 6 = *strongly agree*. Positively coded items included ones such as *I feel that life is very rewarding* and reverse coded items were typified by those such as *I am not particularly optimistic about the future*. The OHQ has been found, after factor analysis with a sample of data from 172 University students, to have satisfactory construct validity by largely possessing a uni-dimensional structure and it also have very good internal consistency with a Cronbach's alpha of .91 (Hills & Argyle, 2002).

### Procedure

Upon clicking on the link to the online survey, participants were given information on the study and an online consent form to complete. To ensure participant anonymity and their right to withdraw from participation if they so wished, participants were required to provide a unique identifier, which could be used to delete a participant's responses at any point up until the analysis stage. After completing the consent section, participants were required to complete the various sections of the survey, after which they were met with a debrief statement that outlined the rationale for the research and pointed participants to resources for support in case of problems relating to video game play. The study was approved by the research team's University's Ethics Committee.

### Design and analysis

The study had a cross-sectional design including correlational data analysis. Multiple regression was used to analyse the predictive capability of five different video game characteristics, happiness, and nine elements of flow in predicting the variance in total Gaming Addiction Scale values.

## Results

A multiple linear regression evaluated the ability of the nine elements of flow, the OHQ, and the five structural gaming characteristics as variables to predict the variance in respondents' GAS total scores. Pre-analysis checks were satisfactory after measuring the degree of multicollinearity in this sample. As can be seen in Table 2, predictor variables were not too highly correlated with each other, with only very strong correlations of .71 and .75 being found for the flow subscale factors. The Variance Inflation Factor (VIF) for the predictor variables ranged from 1.13 to 3.56, which is acceptable as being below the threshold of 10 (Pallant, 2007); likewise, Tolerance levels for each predictor was also satisfactory and ranged from .28 to .88.

Inspection of the correlation matrix revealed the following salient trends, namely that there were low significant positive correlations between GAS levels and social, manipulation/control, and reward features of a game. In terms of relationships with flow, GAS was significantly and positively correlated with a merging of actions and awareness of oneself and also with a distorted sense of time. There was also a moderate inverse correlation between general happiness levels and the sample's GAS experiences. In terms of relationships between predictor variables, the auto-telic experience of playing a video game was weakly but significantly correlated with games that had manipulation/control, narrative and identity, and reward features within them. There were also significant positive correlations between the flow experience of merging actions and awareness of self with various features of a game, including those games with social features and reward features.

Table 3 shows the strength of the predictive relationship with each variable predicting gaming addiction scores. The total variance in game addiction levels explained by this model was 49.2%, (*R^2^* = 0.492, *F*[15, 94] = 6.07, *p* < .05). Three predictor variables were statistically significant predictors of total GAS – social features of a video game, distortion of time perception when playing the game, and levels of happiness; happiness was the strongest predictor (b = –.47), signalling that a one SD unit increase in happiness would predict a .47 SD unit decrease in gaming addiction levels, and vice versa.

**Table 2.** Correlation matrix – Relationship of predictor variables with total gaming addiction scores

	1	2	3	4	5	6	7	8	9	10	11	12	13	14	15	16
1. Total GAS	−															
2. Social features	.24^*^	−														
3. Manipulation/control features	.19^*^	.12	−													
4. Narrative and identity features	.06	.17^*^	.49^*^	−												
5. Presentation features	.12	.55^*^	.34^*^	.41^*^	−											
6. Reward features	.17^*^	.44^*^	.42^*^	.33^*^	.55^*^	−										
7. Balance for challenges & ability	.02	−.01	.10	.09	.05	.08	−									
8. Merging of action & awareness	.21^*^	.28^*^	.01	.10	.21^*^	.24^*^	.26^*^	−								
9. Clearly set goals	.03	.07	.07	.06	.11	.06	.59^*^	.34^*^	−							
10. Unambiguous feedback	−.01	−.05	.06	−.01	.06	.05	.64^*^	.37^*^	.75^*^	−						
11. Concentration on task	.05	.02	.08	−.02	.03	.10	.43^*^	.31^*^	.61^*^	.59^*^	−					
12. Sense of control	.05	−.05	.14	.08	.03	−.01	.59^*^	.37^*^	.68^*^	.71^*^	.65^*^	−				
13. Loss of self-consciousness	−.11	.04	−.02	.10	.03	−.04	−.05	.07	.11	−.02	.06	.16^*^	−			
14. Distortion of time perception	.43^*^	.10	.15	.25^*^	.14	.09	.00	.16^*^	.07	−.08	.14	.07	−.00	−		
15. Autotelic experience	.15	.04	.19^*^	.19^*^	−.01	.18^*^	.53^*^	.31^*^	.59^*^	.51^*^	.63^*^	.66^*^	.14	.18^*^	−	
16. Global happiness	−.50^*^	.00	−.18^*^	.01	.06	.00	.08	.07	.05	.12	.08	.13	−.04	−.12	.02	−
Means	47.10	3.52	5.14	3.64	4.23	5.08	15.63	14.28	16.35	16.35	14.98	16.08	14.70	13.53	15.13	125.70
*SD*	14.04	2.13	1.96	1.69	1.75	1.90	2.84	3.02	2.80	2.84	3.35	2.77	3.79	4.32	3.10	22.02

*Key:* ^*^ indicates significance at *p* < .05; GAS = Game Addiction Scale.

**Table 3.** Summary of multiple regression analysis using the enter method to predict Game Addiction Scale levels

Predictor variable	B	SE B	β
*Structural characteristics*			
Social	1.34^*^	0.63	.20
Manipulation/control	0.19	0.68	.03
Narrative and identity	−0.87	0.78	−.11
Presentation	−0.01	0.85	−.00
Reward	0.28	0.75	.04
*Flow*			
Balance for challenges & ability	−0.14	0.52	−.03
Merging of action & awareness	0.54	0.41	.12
Clearly set goals	−0.68	0.64	−.14
Unambiguous feedback	0.27	0.69	.05
Concentration on task	−0.47	0.47	−.11
Sense of control	0.66	0.68	.13
Loss of self-consciousness	−0.56	0.29	−.15
Distortion of time perception	1.11^*^	0.27	.34
Autotelic experience	0.67	0.54	.15
Global happiness	−0.30^*^	0.05	−.47

*Note: R*^2^ = 0.49; Adjusted *R*^2^ = .41. ^*^ *p* < .05.

## Discussion

By conducting multiple regression analysis on the data collected relating to flow, the structural characteristics of video games, and addiction, the findings provide insights into some of the important factors that may be involved in the development of video game addiction. More specifically, the results demonstrated that three variables were statistically significant predictors of gaming addiction (i.e., the social features of a video game, distortion of time perceptions, and levels of happiness). These all appear to have good face validity in relation to previous findings on video game addiction (Griffiths et al., 2012). These three predictors and the other predictor variables accounted for 49.2% of the variance in Game Addiction Scale levels among the sample; these factors appear to be important factors in explaining how people develop video game addictions.

In relation to happiness, the study showed that the unhappier a player was, the more likely they would have a higher score on the GAS. Given that much of the video game addiction literature shows that video game addicts play as a means of escaping and coping with unpleasant and unwanted aspects of their day-to-day lives (Kuss & Griffiths, 2012a, 2012b), such a finding would appear to make intuitive sense. However, given the cross-sectional nature of the study, the data do not shed light on whether the unhappiness was experienced prior to the game playing (and therefore video game playing was used to counteract the unpleasant feelings) or whether the addictive playing made them feel unhappy (and therefore the video game playing made them forget about how unhappy they were).

The structural characteristic that significantly predicted video game addiction was the social element with increased sociability being associated with higher levels of addictive-like experiences. Structural characteristics that promote sociability are also likely to be deemed as highly rewarding and reinforcing by players, and again any activity that is constantly providing rewarding experiences to the player increases the likelihood of habitual behaviour. The findings in the current study also confirm results obtained by King, Delfabbro and Griffiths (2011) who found video game players with problematic gaming behaviours were significantly more likely, when compared with those with non-problematic game play behaviours, to be engrossed in games with a high social component to them. There are several key dynamics that could be occurring with video games that have a salient socialising characteristic that could make video game addiction more likely. It is probable that an iterative and synergistic process of low levels of happiness, and high levels of certain elements of flow and gaming addiction are blends of experiences that make for gamers seeking out social support systems from within the online game to ameliorate any feelings of isolation.

Indeed, it has been argued in a seminal paper by Selnow (1984) that unhappy, socially isolated gamers may often turn to socialisation through gaming, which then in turn effects a need for spending more time with these ‘electronic friends' in order to feel complete. It is through the social world of the gaming environment that the video game player with pathological tendencies may act out relationships that are somewhat superficial but that are still mutually reinforcing and rewarding; it is often through playing the game at a certain level of skill and the kudos afforded to the gamer through gaming achievements and respect and recognition given by one's gaming companions, that the gamer may attain some form of self-worth. The addictive experiences within the social world of the online video game may thus become mutually reinforcing among many of the players within the gaming community – this dynamic could be particularly problematic for some gamers with a high risk of gaming addiction. With the normalising of behaviours within the video gaming population's social world of devoting prolonged periods of game play in order to succeed, it is little wonder that the norms and values of some of the socially-related video game characteristics may be particularly noxious for someone with a predisposition to video game addiction.

Only one of the nine factors of the flow experience was a significant predictor of gaming addiction – heightened levels of a sense of time being altered during play. This factor may be highly reinforcing and rewarding to video game players, and as such may be an experience that players want to constantly repeat to achieve these positively rewarding psychological experiences. Given that addictive behaviour is essentially about constant rewards (Griffiths, 2005), such a finding again makes intuitive sense. Since flow more generally is widely accepted as a positive optimal psychological experience from engaging in an activity, it makes sense that an activity that is enjoyed so much may in some cases take on an obsessive and/or addictive form. This finding supports the study by Ting-Jui and Chih-Chen (2003) who suggested that flow-inducing activities may lead to addictive behaviours. As also noted earlier, there have been instances within video game addiction research where potential addicts have reported time loss as a negative attribute to gaming (Wood, Griffiths & Parke, 2007). The results from the present study appear to support such a finding.

At present, it is likely that no one type of gaming experience can be associated with higher levels of flow or addiction. In essence, it may not be the phenomenological set of experiences derived from playing a game that is as vital to addiction and flow as perhaps the actual interplay between facets of addiction and flow themselves. Certain, as yet uncovered, facets of gaming experiences may be more crucial to determining a video game player's tendencies to become addicted to a certain game or in getting into a state of flow while playing it. Rather, it is possible that other factors may more crucial to getting into a flow state when gaming, such as the speed with which the gamer can interact with the gaming environment or the need for a focused attentional state (Huang, Chiu, Sung & Farn, 2011).

This study aimed to uncover the potential for assessing common gaming experiences among video game players and also in seeing whether these experiences could be equated with video game addiction. However, as this was only a small-scale pilot study, it is acknowledged that were some limitations, which included issues to do with: the sample size; whether the sample can be seen as representative of the video gamer population and its self-selecting nature; the self-report nature of the data, and that the fact that the cross-sectional design did not make it possible to infer causality.

The study's findings suggest some implications for prevention and treatment of gaming problems. The results suggest that strategies are needed to help game players keep track of time spent during gameplay. Certainly, the extant literature (King, Delfabbro & Griffiths, 2012; King, Delfabbro, Griffiths & Gradisar, 2011, 2012) around the treatment of technology-based addictions, such as internet addiction, has involved recommendations of using a range of therapeutic techniques such as cognitive-behaviour therapy or motivational interviewing to help clients to monitor and cope with unmanageable patterns of behaviour; such techniques could include cognitive-behavioural strategies (e.g. diarising to help the player to be more aware of structural characteristics in a game that has prolonged game play to such an extent that adverse consequences such as conflict, mood modification, and tolerance have resulted). Some responsible gaming companies could introduce features in a game to assist those who may be prone to addictive tendencies from losing track of time while playing; features could be built into a game to remind players to take regular breaks by having subtle ‘pop-up’ messages to inform players of time spent gaming in a single session. Alternatively, as recommended by King, Delfabbro, Griffiths & Gradisar (2012), behavioural strategies such as deploying an alarm clock to set clear parameters for game play time, may also be effective when aiming to interrupt flow as a precursor to addiction. Overall, this pilot study has revealed intriguing results and implications for prevention and treatment of video game addiction, which could benefit from further replication with larger, heterogeneous samples of video game players.
